# Carboxylic Acid Isostere Derivatives of Hydroxypyridinones as Core Scaffolds for Influenza Endonuclease Inhibitors

**DOI:** 10.1021/acsmedchemlett.2c00434

**Published:** 2022-12-09

**Authors:** Ryjul W. Stokes, Alysia J. Kohlbrand, Hyeonglim Seo, Banumathi Sankaran, Johannes Karges, Seth M. Cohen

**Affiliations:** † Department of Chemistry and Biochemistry, University of California, La Jolla, California 92093, United States; § The Berkeley Center for Structural Biology, Advanced Light Source, Lawrence Berkeley National Laboratory, Berkeley, California 94720, United States

**Keywords:** drug discovery, metal-binding pharmacophore, isosteres, influenza endonuclease, medicinal inorganic chemistry

## Abstract

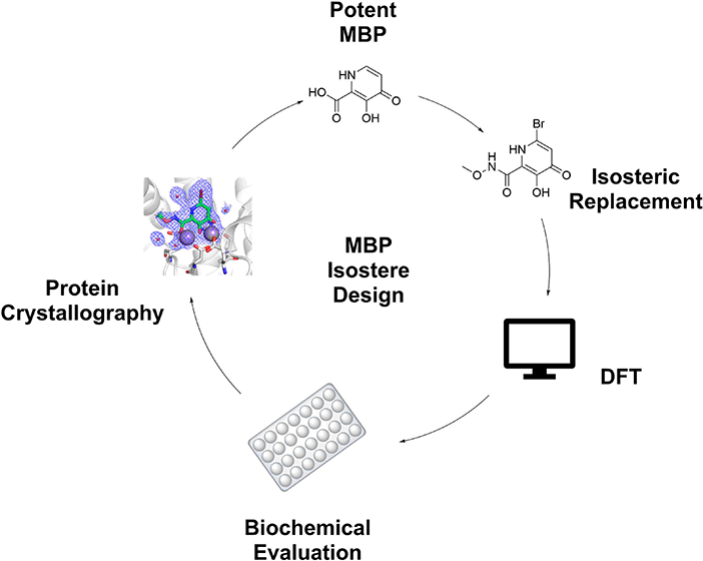

Among the most important influenza virus targets is the RNA-dependent RNA polymerase acidic N-terminal (PA_N_) endonuclease, which is a critical component of the viral replication machinery. To inhibit the activity of this metalloenzyme, small-molecule inhibitors employ metal-binding pharmacophores (MBPs) that coordinate to the dinuclear Mn^2+^ active site. In this study, several metal-binding isosteres (MBIs) were examined where the carboxylic acid moiety of a hydroxypyridinone MBP is replaced with other groups to modulate the physicochemical properties of the compound. MBIs were evaluated for their ability to inhibit PA_N_ using a FRET-based enzymatic assay, and their mode of binding in PA_N_ was determined using X-ray crystallography.

Current estimates suggest that annual influenza epidemics are responsible for up to 650,000 deaths globally.^1^ One recent study concluded that during the 2009 H1N1 pandemic there were ∼61 million cases in the United States alone, resulting in ∼275,000 hospitalizations and 12,000 deaths.^2^ Vaccines are available; however, efficacy depends on the ability to predict antigenic changes, and requires semi-annual re-formulation.^3^ To address acute cases of infection, small-molecule therapeutics have been approved by the U.S. Food and Drug Administration (FDA). Adamantane-based structures that inhibited the matrix protein 2 (M2) ion channel have been used,^4^ but are now largely not prescribed due to resistance.^5,6^ Newer neuraminidase inhibitors are now used,^7^ and resistance to neuraminidase inhibitors remains low; however, emergence of resistance remains a threat to public health.^5,8^ Other small-molecule strategies to address influenza include targeting the hemagglutinin protein^9^ and the RNA-dependent RNA polymerase acidic N-terminal (PA_N_) endonuclease.^10^ PA_N_ represents an especially attractive target due to its role in viral replication, its high conservation, and its lack of a human analog.^11^ FDA approval of baloxavir marboxil, a first-in-class PA_N_ inhibitor, has validated this approach,^12^ but despite its clinical success, resistance against baloxavir has also begun to emerge.^13^

PA_N_ is part of a heterotrimeric RNA-dependent RNA polymerase complex, which is composed of PA, PB1, and PB2 subunits.^14^ Together, they facilitate replication and transcription of the viral genome.^15^ A “cap-snatching” mechanism enables the synthesis of viral mRNA, which can later be translated into viral proteins.^16^ The PA subunit, which enables endonucleolytic cleavage, is composed of a C-terminal domain that is mostly structural and an N-terminal domain that is catalytically active. The N-terminal domain contains a dinuclear Mg^2+^ or Mn^2+^ active site that is highly conserved.^17−19^ Some efforts have used metalloenzyme-focused fragment-based drug discovery (FBDD) to identify potent inhibitors of PA_N_.^20−23^ These FBDD campaigns have identified metal-binding pharmacophores (MBPs) that utilize a triad of oxygen donors, including a carboxylic acid, to bind the metal ions, making these fragments very polar and non-ideal starting points for the development of novel therapeutics. To this end, (bio)isosteric replacement is a strategy that can mitigate pharmacological liabilities.^24^ Herein, the design and experimental evaluation of novel metal-binding isosteres (MBIs) are reported (Figure 1). The MBIs were found to possess good inhibition of PA_N_ endonuclease, with many displaying half-maximal inhibitory concentration (IC_50_) values in the low nanomolar range, comparable to the parent carboxylic acid MBP (compound **1**, Figure 1). Using X-ray crystallography, the binding of these compounds to PA_N_ was elucidated. While many compounds bound as expected (based on prior findings),^21,22^ some isosteres exhibited somewhat unexpected binding interactions. A preliminary analysis of the physical properties, such as calculated log partition coefficient (clogP) and p*K*_a_, shows that the isosteres have tunable properties, potentially circumventing the limitations of the carboxylic acid scaffold.

**Figure 1:**
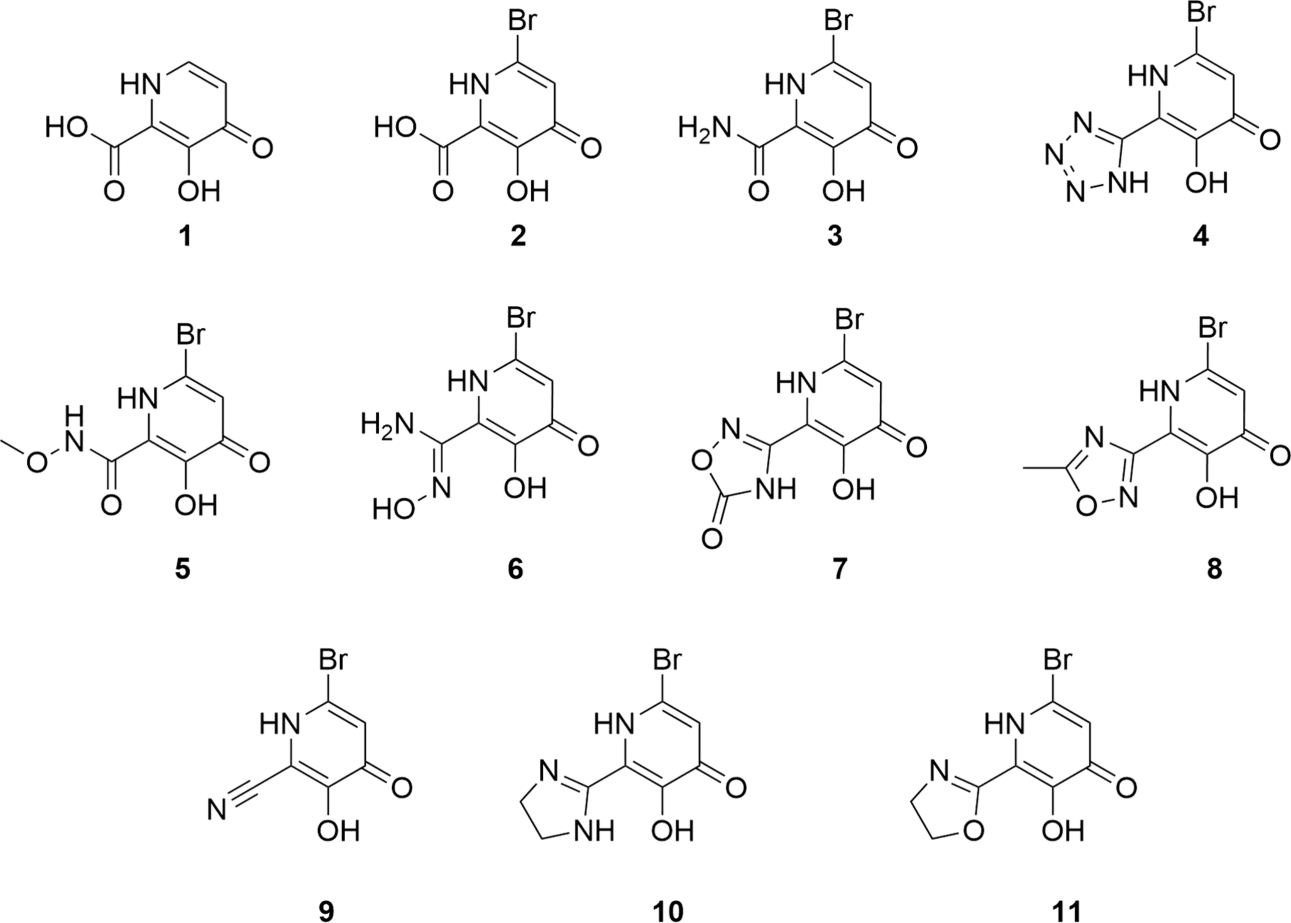
Chemical structures of metal-binding isosteres (MBIs) investigated in this study.

Previous work has focused on the identification of MBP fragments and their evolution into lead-like molecules by FBDD.^20−22^ A carboxylic acid-modified hydroxypyridinone MBP was reported to achieve IC_50_ values as low as ∼17 nM against PA_N_.^21^ Hydroxypyridinones have been utilized in drug discovery campaigns, including for their specific ability to coordinate metal ions.^25^ Despite impressive activity, this MBP (and elaborated inhibitors derived from it) was found to be only moderately active in influenza-infected cells. This disparity between antiviral activity and enzymatic inhibition is likely due to low membrane permeability originating from the high polarity of these molecules.^19^ Additionally, it is well-known that carboxylic acid moieties are associated with pharmacological liabilities, which can limit their use in drug development.^26−28^ The carboxylic acid functional group can be replaced by a different moiety with similar biochemical interactions, but different physiochemical properties, via isosteric replacement to try to address possible shortcomings of the carboxylic acid-containing MBP **1** (Figure 1).^24,29^

Nine MBIs (**3**–**11**) of hydroxypyridinone MBP **1** were synthesized, with isosteric replacements for the carboxylic acid moiety installed (Figure 1). These MBIs also possess a bromine atom in the 6-position of the ring to aid in crystallography (i.e., via heavy-atom substitution and anomalous scattering) and to serve as a synthetic handle for future compound elaboration.^21^ To account for the electronic and lipophilic effects of adding this bromine substituent, a direct analog of **1** was synthesized that contains a bromine atom (**2**, Figure 1). Table S1 lists the calculated clogP values of these fragments compared to analogues substituted with -H, -CH_3_, or -Ph groups.

## Compound Synthesis

**Scheme 1:**
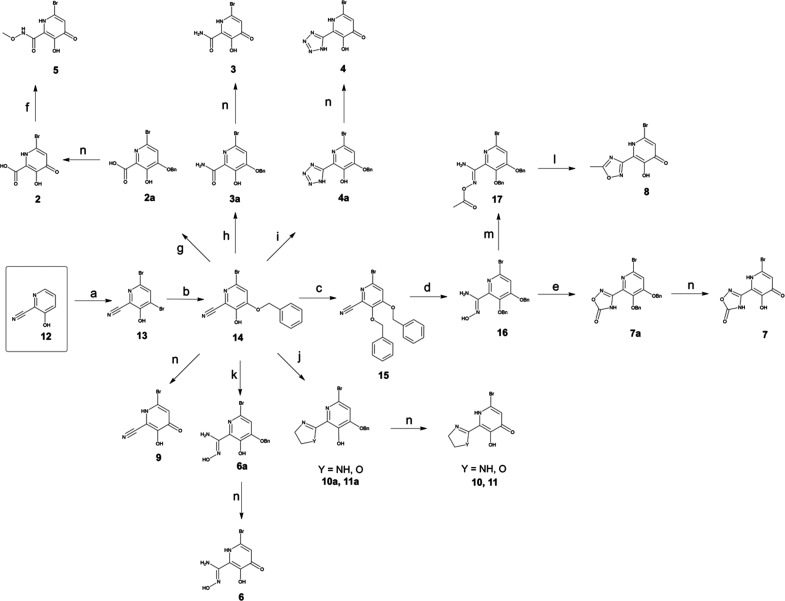
Synthesis of MBIs Reagents and conditions: (a) NaOAc, H_2_O:MeOH, Br_2_, 0 °C to 25 °C; (b) NaH, BnOH, DMSO, 60 °C, 18 h; (c) BnBr, K_2_CO_3_, DMF, 45 °C, 19 h; (d) HONH_2_·HCl, NEt_3_, EtOH, 50 °C, 15 h; (e) CDI, NEt_3_, DMF, 80 °C, 8 h; (f) i. SOCl_2_, DCM, reflux, 12 h. ii. Methoxyamine hydrochloride, NEt_3_, DCM, −78 °C to 40 °C, 48 h; (g) NaOH, EtOH, 100 °C, 20 h; (h) KOH, EtOH:H_2_O, 100 °C, 16 h; (i) NaN_3_, NH_4_Cl, DMF, 115 °C, 4 h; (j) ethane-1,2-diamine or 2-aminoethan-1-ol, ZnCl_2_, toluene, 90 °C to 130 °C, 16–20 h; (k) HONH_2_·HCl, NEt_3_, EtOH, 50 °C 1 h; (l) HCl:AcOH:TFA (5:5:1), 50 °C, 48 h; (m) Ac_2_O, DBU, THF, 0 °C to 80 °C, 8 h; (n) HCl, 25 °C to 100 °C, 12–48 h.

All bromine-containing compounds (**2**–**11**) were synthesized from precursor **12**, 3-hydroxypicolinonitrile (Scheme 1). Commercially available **12** can be dibrominated selectively in the 4- and 6-positions upon treatment with elemental bromine to afford **13** in high yield. An oxygen atom that will later participate as a Lewis base to bind the Mn^2+^ center can be introduced via S_N_Ar chemistry. This nucleophilic addition results in the selective introduction of benzyl alcohol in the 4-position to afford intermediate **14** in good yield. Compound **14** serves as a crucial intermediate for the compounds evaluated in this study. Hydrolysis of **14** under basic conditions yields the benzyl-protected compounds **2a** and **3a**, which can be deprotected under acidic conditions to afford compounds **2** and **3**, the acid- and amide-functionalized molecules, respectively. Compound **2** can be further functionalized to incorporate an *N*-methoxycarboxamide group (**5**) by conversion to the acid chloride and subsequent treatment with methoxyamine hydrochloride. A click reaction between compound **14** and sodium azide results in the formation of tetrazole **4a**, a commonly employed carboxylic acid isostere, which can be deprotected to afford the tridentate MBI **4**. Treatment of compound **14** with zinc chloride and ethanolamine or ethylenediamine results in the partially saturated heterocycles **10a** and **11a**. Saturated heterocycles can be advantageous as they tend to have higher lipophilicity, solubility, and three-dimensionality than their unsaturated counterparts.^30^ Deprotection of these compounds with HCl results in the formation of the partially saturated imidazoline (**10**) and oxazoline (**11**). The nitrile group in compound **14** can also be transformed into an *N*-hydroxyamidine functional group when treated with hydroxylamine hydrochloride and triethylamine, followed by debenzylation to afford compound **6**. Finally, simple deprotection of compound **14** under acidic conditions results in the nitrile-containing MBI **9**. In some cases, protection of the phenol in compound **14** was beneficial. Treatment of **14** with benzyl bromide and potassium carbonate resulted in the formation of **15** in good yield. The protected *N*-hydroxyamide intermediate was synthesized using conditions similar to those used for **6a**, though longer reaction times were employed. Compound **16** can be acylated upon the addition of acetic anhydride to afford compound **17**, which when exposed to heat and a mixture of hydrochloric acid, acetic acid, and trifluoroacetic acid undergoes both debenzylation and an acid-promoted cyclization to afford oxadiazole **8** in a single step. Additionally, treatment of compound **16** with carbonyldiimidazole (CDI) results in the formation of the oxadiazolone-functionalized molecule **7a**, which can be deprotected to afford compound **7**.

Previous studies have correlated the acidity of the phenolic proton (p*K*_a_) of the hydroxypyridinone fragment with the ability to inhibit the activity of PA_N_.^22^ In addition, changes in p*K*_a_ can be telling indicators of changes in the electronic structure of the heterocycle. Accordingly, p*K*_a_ values of the MBIs were calculated to probe the electronic differences introduced by the isostere groups. The p*K*_a_ value of the phenolic proton was estimated using density functional theory (DFT) calculations. More precisely, the energetic states within a thermodynamic cycle of the proton-transfer reaction were calculated, and the resulting free energy was correlated to the p*K*_a_ values as has been previously described (see Supporting Information for details).^31^ Compound **2** was predicted to have a p*K*_a_ value of 9.4. Using a pH-metric titration, the measured p*K*_a_ value of compound **2** was measured at 9.79 ± 0.01, showing reasonable agreement with the DFT calculation. The isosteric replacement of this functional group was identified as an influencing factor of the acidity of the phenolic proton (a full list of predicted and several additional measured p*K*_a_ values is provided in Table S2). The isostere modification of the carbonyl group gave calculated p*K*_a_ values ranging from 8.1 to 12.6 (Table S2), indicating that changes in the 2-position of the hydroxypyridinone ring can have large effects on the electronics of the ring system, and is a useful means to tune the electronic properties of the ring.

**Figure 2:**
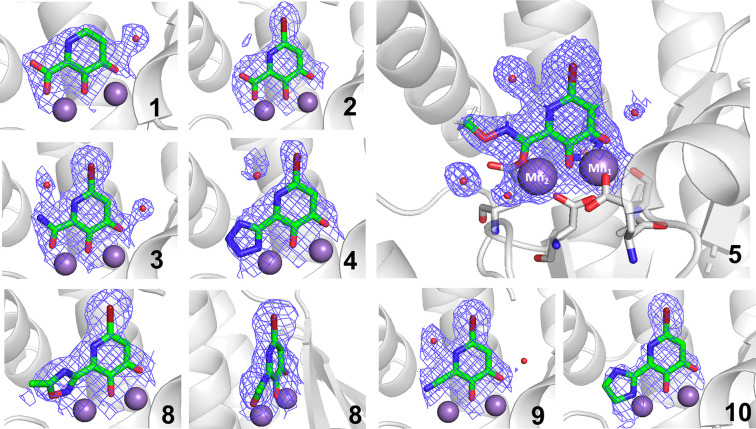
Co-crystal structures of PA_N_ endonuclease with compounds **1**–**5** and **8**−**10**. The large panel depicts MBI **5** coordinating Mn_1_ through the exocyclic carbonyl oxygen atom, Mn_2_ through the carboxylate group, and the hydroxyl donor bridging between Mn_1_ and Mn_2_. The protein backbone is shown as a gray cartoon. Mn^2+^ ions and water molecules are shown as purple and red spheres, respectively. The structure with compound **8** is shown from two perspectives. Residues coordinating the Mn^2+^ ions are shown in sticks. Atom colors are as follow: carbon (green MBI, gray protein), oxygen (red), nitrogen (blue), bromine (dark red). The electron density of each MBI and the Mn^2+^ ions is displayed as a blue mesh. Mesh is 2*F*_o_ – *F*_c_ contoured at 2σ.

To determine the binding mode of the MBIs in the PA_N_ active site, several compounds were crystallized with the enzyme for structure determination. Tables of collection and refinement statistics are provided in the Supporting Information (Tables S3, S4). In native PA_N_, the Mn^2+^ cations are coordinated by His41, Glu80, Asp108, Glu 119, and Ile120 and five water molecules, producing an octahedral coordination geometry at each metal center. As anticipated, the addition of a bromine atom to the MBIs produced no apparent change in the binding to the active site metal ions (compare structures of **1** and **2**, Figure 2) and aided in ligand placement within the electron density map. Both compounds **1** and **2** coordinated by bridging the metal ions through the phenolate donor. This provides a triad of oxygen donor atoms (including oxygen donor atoms from the ketone and carboxylic acid groups), creating a 5-membered chelate ring at Mn_1_ and a 6-membered chelate ring at Mn_2_. Compounds **1** and **2** also displayed hydrogen bonding between the ketone donor from the hydroxypyridinone ring and Lys134.^32^

Previous crystallographic studies have focused on compounds containing a triad of chelating oxygen atoms that bind in a tridentate fashion, displacing three of the coordinating water molecules.^21,22^ Compounds **1**, **2**, **3**, and **5** present the same triad and display similar binding poses, coordinating Mn_1_ through the ketone and Mn_2_ through the carboxylate, with the hydroxyl group bridging Mn_1_ and Mn_2_ (Figure 2).^21^ The binding and inhibitory activity (vide infra) of these compounds are comparable to those of the parent MBP **1**. The average distance from Mn_2_ to the carboxylic acid isosteric group in each of these structures is 2.2 Å. A list of metal–donor atom distances is provided in Table S5. Interestingly, several nitrogen-containing isosteres were found to have a bridging water molecule between the nitrogen of the hydroxypyridinone core and a nitrogen from the isosteric groups (see compounds **3**, **4**, **5**, and **9**, Figure 2). This interaction is reminiscent of the carbamoylpyridine bicycle in Baloxavir,^19^ but creates a cyclized, rigid structure by hydrogen bonding with water, rather than a synthetic, covalent ring structure. This pseudo-five-membered ring may be a useful in future inhibitor design, as it provides conformational stabilization without the necessity to form a bicycle, affording additional synthetic opportunities to develop interactions in the pocket toward Tyr24 and α-helix 2.

Among structures, compounds **4**, **8**, **9**, and **10** break with the chelating oxygen triad motif by replacing the carboxylic acid oxygen with a nitrogen atom. As expected, Mn_1_ is bound between the phenol and the ketone of the hydroxypyridinone core, but unlike previous MBIs with a third oxygen donor, rotations of varying degrees were observed with the heterocyclic MBIs when bound to Mn_2_. Interestingly, the oxadiazolone ring of compound **8** is noticeably rotated out of the plane of the hydroxypyridinone core. A possible explanation for the rotation is the driving force to maintain an octahedral coordination geometry at both metal centers. To preserve an acceptable angle/distance between the metal and the donor atom at both metal sites, a rotation of the heterocyclic isostere may occur, the metal–ligand distances may be adjusted, or a combination thereof. To further examine this hypothesis, octahedral distortion parameters ζ and Σ were calculated for compound **8** (relative to Mn_2_) and compared with the distortion parameters of compound **3** and the ligand-free structure, which presented the most ideal octahedral geometries. To calculate and visualize changes in octahedral geometry, Octadist,^33^ an octahedral distortion analysis software, was used (Figure S1, Table S6). The ζ and Σ values are general octahedral distortion parameters used to characterize deviations from ideal octahedral geometry and are used here to evaluate the isostere binding geometry to Mn_2_.^33^ The value of ζ is the average of the sum of the deviation of 6 unique metal–ligand bond lengths around the central metal atom (*d*_i_) from the average value (*d*_mean_).^34^ The value of Σ is the sum of the deviation of 12 unique *cis* ligand–metal–ligand angles (ϕ_*i*_) from 90°.^35,36^ These distortion values were calculated for compound **8**, although it should be noted that these values do not take into account the resolution of the crystal structure, and should be considered accordingly. The value for ζ is very small, 0.574 Å, suggesting only very small changes to ligand–metal distances for each of the six donor atoms. The Σ value is also rather small, 90.81°, with the largest deviations from 90° resultant from contributions of the more rigid angles associated with Glu80 and Asp108. When these distortion parameters are calculated for a structure of compound **8** that is forced into an idealized co-planar ligand geometry, the ζ value increases to 0.606 Å and Σ increases to 98.347°, suggesting that the planar MBI structure enforces an inferior octahedral geometry at the metal centers compared to the experimental structure. Comparing both the rotated and planar structures of compound **8** to those of compound **3** (0.357 Å, 46.669°) and the ligand-free structure (bound water, 0.685 Å, 44.571°), both conformations of compound **8** exhibit poorer octahedral character.

The tetrazole in compound **4** displays a slight twist, perhaps due to the same reason as compound **8**, though the rotation appears less pronounced. Interestingly, instead of incorporating a more distinct twist, this structure adopts different metal–ligand binding distances. The distance between Mn_1_ and the ketone functional group is 2.3 Å, while the distance between the tetrazole and Mn_2_ is 1.8 Å, both the longest and shortest metal–donor distances observed among these inhibitors, respectively. The average distance from the metal cation in each nitrogen-donating structure is 2.2 Å, matching the donor–metal distance in oxygen triad MBIs, furthering the argument that the rotations of compounds **4** and **8** result from attempting to optimize metal-binding interactions.

Overall, MBIs following the same donor oxygen triad displayed metal-binding activity and binding poses similar to those of previously reported structures. MBIs presenting a nitrogen donor atom instead of the carboxylate oxygen have more variability in metal-binding behavior and pose. Heterocyclic nitrogen MBIs were found to have varying degrees of internal ligand rotation, most notably with compound **8**, which is most likely explained by the need to optimize the octahedral coordination geometry at the Mn_2_ metal center.

To determine the activity of these molecules against PA_N_, MBIs were evaluated using a fluorescence-quenching-based nuclease activity assay (see Supporting Information for details).^37^ Using this assay, dose–response curves were obtained to determine the half-maximal inhibitory concentration (IC_50_) values of these compounds. Importantly, IC_50_ values are strongly dependent on the buffer, pH, enzyme concentration, and substrate used, and as such may vary somewhat with assay conditions.^38^ Here, a protein concentration of 25 nM was employed.^17^ As previously identified hydroxypyridinones have been shown to be tight binders of PA_N_,^21^ it is expected that molecules with good metal-binding motifs will demonstrate low IC_50_ values under these assay conditions.^39^ The results of the dose–response experiment are shown in Table 1.

**Table 1:**
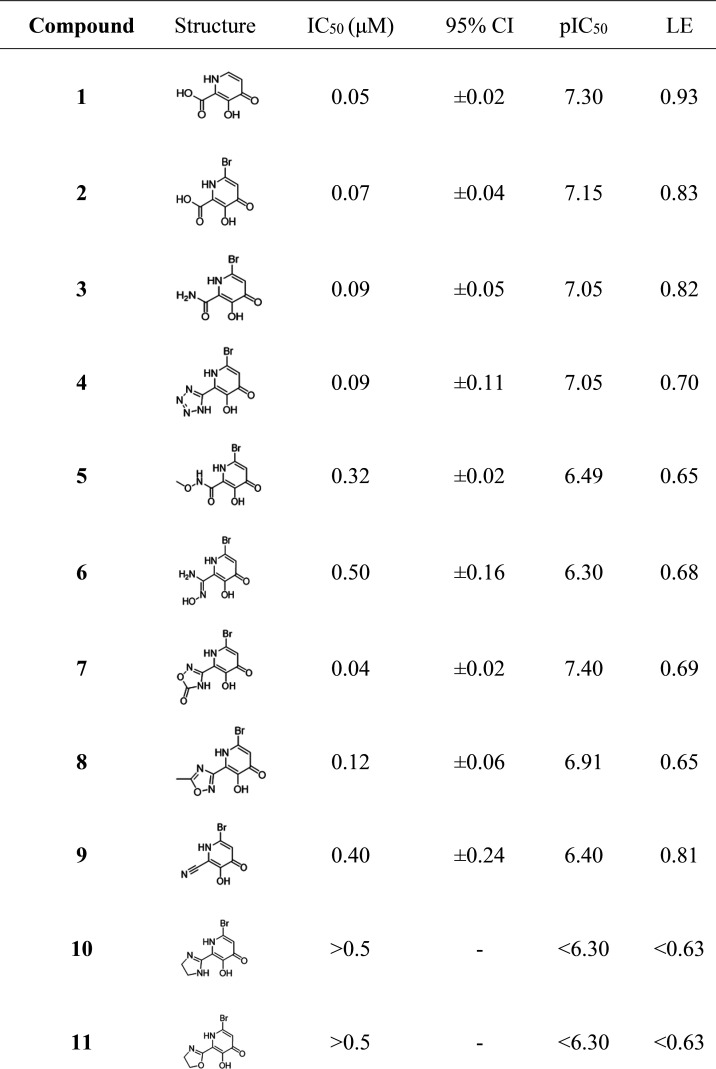
Half-Maximal Inhibitory Concentration (IC_50_), 95% Confidence Interval (95% CI), log IC_50_ (pIC_50_), and Ligand Efficiency (LE) of Compounds **1**–**11** against PA_N_ Endonuclease

Compound **2** was used as a control to determine whether the addition of a bromine atom negatively affected the metal-binding ability of the MBP. Compounds **1** and **2** showed similar inhibition activity (Table 1), indicating that the bromide did not impact metal-binding relative to the parent compound. Both compounds display high binding energy per atom (ligand efficiency, LE) (Table 1). Despite conversion from a potentially anionic carboxylate to a neutral carboxamide, compound **3** showed little difference in activity when compared with compounds **1** or **2**. Compound **4** showed activity comparable to that of the parent structure, possibly due to shorter metal-ligand bond distances (Table S5). This is perhaps unsurprising, as tetrazoles have been widely investigated as isosteres for carboxylic acids.^40^ Compound **5** demonstrated moderate inhibition, suggesting somewhat poorer metal-binding ability, perhaps due to the electron-withdrawing nature of the methoxy group. Compound **7** demonstrated the most potent IC_50_ value of the evaluated compounds; however, a crystal structure was not obtained. Compound **8**, functionalized with a methyl-oxadiazole, showed good inhibition, indicating that the rotation of the heterocycle may contribute to improved metal-binding via optimizing the metal–ligand geometry. While compound **8** does bind favorably, it demonstrates higher distortion parameters than compound **3**, possibly accounting for the decreased activity of **8** compared to **3**. Compound **9** showed relatively poor inhibition, due to the loss of a strong interaction with Mn_2_, which cannot be provided by the nitrile isostere in this context. Previous structures have shown bidentate hydroxypyridinones coordinating to Mn_1_, with the ketone bridging the Mn_1_ and Mn_2_ metals with the hydroxyl group bound to only Mn_1_.^17,21^ Consistent with every structure reported in this study, compound **9** bridges the metal ions through the anionic phenolic group, while the ketone group interacts with Mn_2_ and Lys134. This result indicates that even with poorer-binding isosteres like **9**, the introduction of a substituent in the 2-position of the hydroxypyridine ring drives the MBI to coordinate in such a way that bridges the metals with the phenolic group, which is a stronger donor than the ketone and should give better overall affinity. Finally, the saturated heterocycle isosteres **10** and **11** showed markedly poorer inhibition than the parent MBI, possibly due to the softer nature of the heterocyclic donor nitrogen atoms, resulting in poorer electron donation to Mn_2_.

In summary, MBIs of the highly active hydroxypyridinone scaffold were designed to replace the carboxylic acid group and retain inhibition of PA_N_ endonuclease. A variety of fragments with isosteric replacements were synthesized via a common intermediate and evaluated in a FRET-based enzymatic assay. Many of these molecules retain inhibitory activity comparable to that of the parent carboxylic acid fragment. These molecules were further evaluated crystallographically to improve our understanding of how the structural and metal-binding properties are affected by replacement of the carboxylic acid group. This study shows that MBIs may be useful in the development of small-molecule therapeutics of influenza PA_N_ and other related nucleic-acid-processing viral metalloenzymes.^41,42^

## Supporting Information Available

Computational prediction of p*K*_a_, comparative predictions of clogP, predicted phenolic p*K*_a_ values, protein expression and purification, protein crystallography; protein crystallography tables, metal-binding distances, octahedral distortion parameters, octahedral distortion, representative HPLC traces, and lipophilic ligand efficiencies (LLE) (PDF)
SMILES strings formula (CSV)

## Abbreviations

clogP: calculated log partition coefficient
CDI: carbonyldiimidazole
DBU: 1,8-diazabicyclo(5.4.0)undec-7-ene
DCM: dichloromethane
DFT: density functional theory
DMF: dimethylformamide
EtOAc: ethyl acetate
FDA: U.S. Food and Drug Administration
IC_50_: half-maximal inhibitory concentration
HAC: heavy atom count
Hex: hexanes
LE: ligand efficiency
M2: matrix protein 2
MBI: metal-binding isostere
MeOH: methanol
MBP: metal-binding pharmacophore
PAN: N-terminal domain of the RNA-dependent RNA polymerase PA subunit
S_N_Ar: nucleophilic aromatic substitution
SAR: structure–activity relationship

## References

[ref1] Iuliano A D.; Roguski K. M; Chang H. H; Muscatello D. J; Palekar R.; Tempia S.; Cohen C.; Gran J. M.; Schanzer D.; Cowling B. J; Wu P.; Kyncl J.; Ang L. W.; Park M.; Redlberger-Fritz M.; Yu H.; Espenhain L.; Krishnan A.; Emukule G.; van Asten L.; Pereira da Silva S.; Aungkulanon S.; Buchholz U.; Widdowson M.-A.; Bresee J. S; Azziz-Baumgartner E.; Cheng P.-Y.; Dawood F.; Foppa I.; Olsen S.; Haber M.; Jeffers C.; MacIntyre C R.; Newall A. T; Wood J. G; Kundi M.; Popow-Kraupp T.; Ahmed M.; Rahman M.; Marinho F.; Sotomayor Proschle C V.; Vergara Mallegas N.; Luzhao F.; Sa L.; Barbosa-Ramirez J.; Sanchez D. M.; Gomez L. A.; Vargas X. B.; Acosta Herrera a.; Llanes M. J.; Fischer T. Køl.; Krause T. G.; Mølbak K.; Nielsen J.; Trebbien R.; Bruno A.; Ojeda J.; Ramos H.; an der Heiden M.; del Carmen Castillo Signor L.; Serrano C. E.; Bhardwaj R.; Chadha M.; Narayan V.; Kosen S.; Bromberg M.; Glatman-Freedman A.; Kaufman Z.; Arima Y.; Oishi K.; Chaves S.; Nyawanda B.; Al-Jarallah R. A.; Kuri-Morales P. A; Matus C. R.; Corona M. E. J.; Burmaa A.; Darmaa O.; Obtel M.; Cherkaoui I.; van den Wijngaard C. C; van der Hoek W.; Baker M.; Bandaranayake D.; Bissielo A.; Huang S.; Lopez L.; Newbern C.; Flem E.; Grøneng G. M; Hauge S.; de Cosio F. G; de Molto Y.; Castillo L. M.; Cabello M. A.; von Horoch M.; Medina Osis J.; Machado A.; Nunes B.; Rodrigues A. P.; Rodrigues E.; Calomfirescu C.; Lupulescu E.; Popescu R.; Popovici O.; Bogdanovic D.; Kostic M.; Lazarevic K.; Milosevic Z.; Tiodorovic B.; Chen M.; Cutter J.; Lee V.; Lin R.; Ma S.; Cohen A. L; Treurnicht F.; Kim W. J.; Delgado-Sanz C.; de mateo Ontanon S.; Larrauri A.; Leon I. L.; Vallejo F.; Born R.; Junker C.; Koch D.; Chuang J.-H.; Huang W.-T.; Kuo H.-W.; Tsai Y.-C.; Bundhamcharoen K.; Chittaganpitch M.; Green H. K; Pebody R.; Goni N.; Chiparelli H.; Brammer L.; Mustaquim D.; et al. Estimates of global seasonal influenza-associated respiratory mortality: a modelling study. Lancet 2018, 391, 1285–1300. 10.1016/S0140-6736(17)33293-2. 29248255 PMC5935243

[ref2] Shrestha S. S.; Swerdlow D. L.; Borse R. H.; Prabhu V. S.; Finelli L.; Atkins C. Y.; Owusu-Edusei K.; Bell B.; Mead P. S.; Biggerstaff M.; Brammer L.; Davidson H.; Jernigan D.; Jhung M. A.; Kamimoto L. A.; Merlin T. L.; Nowell M.; Redd S. C.; Reed C.; Schuchat A.; Meltzer M. I. Estimating the Burden of 2009 Pandemic Influenza A (H1N1) in the United States (April 2009–April 2010). Clin. Infect. Dis. 2011, 52, S75–S82. 10.1093/cid/ciq012. 21342903

[ref3] Lewnard J. A.; Cobey S. Immune History and Influenza Vaccine Effectiveness. Vaccines 2018, 6, 28 10.3390/vaccines6020028. 29883414 PMC6027411

[ref4] Pielak R. M.; Chou J. J. Influenza M2 proton channels. Biochim. Biophys. Acta, Biomembr. 2011, 1808, 522–529. 10.1016/j.bbamem.2010.04.015. PMC3108042 20451491

[ref5] Principi N.; Camilloni B.; Alunno A.; Polinori I.; Argentiero A.; Esposito S. Drugs for Influenza Treatment: Is There Significant News?. Front. Med. 2019, 6, 109 10.3389/fmed.2019.00109. PMC6546914 31192211

[ref6] Bright R. A.; Shay D. K.; Shu B.; Cox N. J.; Klimov A. I. Adamantane Resistance Among Influenza A Viruses Isolated Early During the 2005–2006 Influenza Season in the United States. JAMA 2006, 295, 891–894. 10.1001/jama.295.8.joc60020. 16456087

[ref7] Moscona A. Neuraminidase Inhibitors for Influenza. N. Engl. J. Med. 2005, 353, 1363–1373. 10.1056/NEJMra050740. 16192481

[ref8] Kim H. M.; Lee N.; Kim M.-S.; Kang C.; Chung Y.-S. Characterization of neuraminidase inhibitor-resistant influenza virus isolates from immunocompromised patients in the Republic of Korea. Virol. J. 2020, 17, 94 10.1186/s12985-020-01375-1. 32631440 PMC7338124

[ref9] Sriwilaijaroen N.; Suzuki Y. Molecular basis of the structure and function of H1 hemagglutinin of influenza virus. Proc. Jpn. Acad., Ser. B 2012, 88, 226–249. 10.2183/pjab.88.226. 22728439 PMC3410141

[ref10] Yuan P.; Bartlam M.; Lou Z.; Chen S.; Zhou J.; He X.; Lv Z.; Ge R.; Li X.; Deng T.; Fodor E.; Rao Z.; Liu Y. Crystal structure of an avian influenza polymerase PAN reveals an endonuclease active site. Nature 2009, 458, 909–913. 10.1038/nature07720. 19194458

[ref11] Monod A.; Swale C.; Tarus B.; Tissot A.; Delmas B.; Ruigrok R. W.; Crépin T.; Slama-Schwok A. Learning from structure-based drug design and new antivirals targeting the ribonucleoprotein complex for the treatment of influenza. Expert Opin. Drug Discovery 2015, 10, 345–371. 10.1517/17460441.2015.1019859. 25792362

[ref12] Ng K. E. Xofluza (Baloxavir Marboxil) for the Treatment Of Acute Uncomplicated Influenza. P&T 2019, 44, 9–11. 30675086 PMC6336199

[ref13] Checkmahomed L.; M’Hamdi Z.; Carbonneau J.; Venable M. C.; Baz M.; Abed Y.; Boivin G. Impact of the Baloxavir-Resistant Polymerase Acid I38T Substitution on the Fitness of Contemporary Influenza A(H1N1)pdm09 and A(H3N2) Strains. J. Infect. Dis. 2020, 221, 63–70. 10.1093/infdis/jiz418. 31419295 PMC6910874

[ref14] Resa-Infante P.; Jorba N.; Coloma R.; Ortin J. The influenza virus RNA synthesis machine: advances in its structure and function. RNA Biol. 2011, 8, 207–215. 10.4161/rna.8.2.14513. 21358279 PMC3127100

[ref15] Hutchinson E. C.; Fodor E. Transport of the influenza virus genome from nucleus to nucleus. Viruses 2013, 5, 2424–2446. 10.3390/v5102424. 24104053 PMC3814596

[ref16] Dias A.; Bouvier D.; Crépin T.; McCarthy A. A.; Hart D. J.; Baudin F.; Cusack S.; Ruigrok R. W. H. The cap-snatching endonuclease of influenza virus polymerase resides in the PA subunit. Nature 2009, 458, 914–918. 10.1038/nature07745. 19194459

[ref17] Bauman J. D.; Patel D.; Baker S. F.; Vijayan R. S. K.; Xiang A.; Parhi A. K.; Martínez-Sobrido L.; LaVoie E. J.; Das K.; Arnold E. Crystallographic Fragment Screening and Structure-Based Optimization Yields a New Class of Influenza Endonuclease Inhibitors. ACS Chem. Biol. 2013, 8, 2501–2508. 10.1021/cb400400j. 23978130 PMC3928712

[ref18] Jones J. C.; Marathe B. M.; Lerner C.; Kreis L.; Gasser R.; Pascua P. N. Q.; Najera I.; Govorkova E. A. A Novel Endonuclease Inhibitor Exhibits Broad-Spectrum Anti-Influenza Virus Activity In Vitro. Antimicrob. Agents Chemother. 2016, 60, 5504–5514. 10.1128/AAC.00888-16. 27381402 PMC4997863

[ref19] Miyagawa M.; Akiyama T.; Taoda Y.; Takaya K.; Takahashi-Kageyama C.; Tomita K.; Yasuo K.; Hattori K.; Shano S.; Yoshida R.; Shishido T.; Yoshinaga T.; Sato A.; Kawai M. Synthesis and SAR Study of Carbamoyl Pyridone Bicycle Derivatives as Potent Inhibitors of Influenza Cap-dependent Endonuclease. J. Med. Chem. 2019, 62, 8101–8114. 10.1021/acs.jmedchem.9b00861. 31386363

[ref20] Credille C. V.; Chen Y.; Cohen S. M. Fragment-Based Identification of Influenza Endonuclease Inhibitors. J. Med. Chem. 2016, 59, 6444–6454. 10.1021/acs.jmedchem.6b00628. 27291165 PMC4948595

[ref21] Credille C. V.; Morrison C. N.; Stokes R. W.; Dick B. L.; Feng Y.; Sun J.; Chen Y.; Cohen S. M. SAR Exploration of Tight-Binding Inhibitors of Influenza Virus PA Endonuclease. J. Med. Chem. 2019, 62, 9438–9449. 10.1021/acs.jmedchem.9b00747. 31536340

[ref22] Credille C. V.; Dick B. L.; Morrison C. N.; Stokes R. W.; Adamek R. N.; Wu N. C.; Wilson I. A.; Cohen S. M. Structure–Activity Relationships in Metal-Binding Pharmacophores for Influenza Endonuclease. J. Med. Chem. 2018, 61, 10206–10217. 10.1021/acs.jmedchem.8b01363. 30351002 PMC6249039

[ref23] Karges J.; Stokes R. W.; Cohen S. M. Photorelease of a metal-binding pharmacophore from a Ru(ii) polypyridine complex. Dalton Trans. 2021, 50, 2757–2765. 10.1039/D0DT04290K. 33564808 PMC7944940

[ref24] Lassalas P.; Gay B.; Lasfargeas C.; James M. J.; Tran V.; Vijayendran K. G.; Brunden K. R.; Kozlowski M. C.; Thomas C. J.; Smith A. B.; Huryn D. M.; Ballatore C. Structure Property Relationships of Carboxylic Acid Isosteres. J. Med. Chem. 2016, 59, 3183–3203. 10.1021/acs.jmedchem.5b01963. 26967507 PMC4833640

[ref25] Santos M. A.; Marques S. M.; Chaves S. Hydroxypyridinones as “privileged” chelating structures for the design of medicinal drugs. Coord. Chem. Rev. 2012, 256 (1), 240–259. 10.1016/j.ccr.2011.08.008.

[ref26] Lassila T.; Hokkanen J.; Aatsinki S.-M.; Mattila S.; Turpeinen M.; Tolonen A. Toxicity of Carboxylic Acid-Containing Drugs: The Role of Acyl Migration and CoA Conjugation Investigated. Chem. Res. Toxicol. 2015, 28, 2292–2303. 10.1021/acs.chemrestox.5b00315. 26558897

[ref27] Pajouhesh H.; Lenz G. R. Medicinal chemical properties of successful central nervous system drugs. NeuroRx 2005, 2, 541–553. 10.1602/neurorx.2.4.541. 16489364 PMC1201314

[ref28] Dick B. L.; Cohen S. M. Metal-Binding Isosteres as New Scaffolds for Metalloenzyme Inhibitors. Inorg. Chem. 2018, 57, 9538–9543. 10.1021/acs.inorgchem.8b01632. 30009599 PMC6289299

[ref29] Patani G. A.; LaVoie E. J. Bioisosterism: A Rational Approach in Drug Design. Chem. Rev. 1996, 96, 3147–3176. 10.1021/cr950066q. 11848856

[ref30] Marson C. M. Saturated Heterocycles with Applications in Medicinal Chemistry. In Advances in Heterocyclic Chemistry; Scriven E. F. V., Ramsden C. A., Eds.; Academic Press: 2017; Vol. 121, Chap. 2, 13–33.

[ref31] Pliego J. R. Thermodynamic cycles and the calculation of pKa. Chem. Phys. Lett. 2003, 367 (1), 145–149. 10.1016/S0009-2614(02)01686-X.

[ref32] Zhang C.; Xiang J.; Xie Q.; Zhao J.; Zhang H.; Huang E.; Shaw P.; Liu X.; Hu C. Identification of Influenza PAN Endonuclease Inhibitors via 3D-QSAR Modeling and Docking-Based Virtual Screening. Molecules 2021, 26, 7129 10.3390/molecules26237129. 34885710 PMC8659138

[ref33] Ketkaew R.; Tantirungrotechai Y.; Harding P.; Chastanet G.; Guionneau P.; Marchivie M.; Harding D. J. OctaDist: a tool for calculating distortion parameters in spin crossover and coordination complexes. Dalton Trans. 2021, 50, 1086–1096. 10.1039/D0DT03988H. 33367357

[ref34] Buron-Le Cointe M.; Hébert J.; Baldé C.; Moisan N.; Toupet L.; Guionneau P.; Létard J. F.; Freysz E.; Cailleau H.; Collet E. Intermolecular control of thermoswitching and photoswitching phenomena in two spin-crossover polymorphs. Phys. Rev. B 2012, 85, 064114 10.1103/PhysRevB.85.064114.

[ref35] Drew M. G. B.; Harding C. J.; McKee V.; Morgan G. G.; Nelson J. Geometric control of manganese redox state. J. Chem. Soc., Chem. Commun. 1995, 1035–1038. 10.1039/c39950001035.

[ref36] Guionneau P.; Brigouleix C.; Barrans Y.; Goeta A. E.; Létard J.-F.; Howard J. A. K.; Gaultier J.; Chasseau D. High pressure and very low temperature effects on the crystal structures of some iron(II) complexes. C. R. Acad. Sci., Ser. IIc: Chim. 2001, 4, 161–171. 10.1016/S1387-1609(00)01193-2.

[ref37] Baughman B. M.; Jake Slavish P.; DuBois R. M.; Boyd V. A.; White S. W.; Webb T. R. Identification of Influenza Endonuclease Inhibitors Using a Novel Fluorescence Polarization Assay. ACS Chem. Biol. 2012, 7, 526–534. 10.1021/cb200439z. 22211528 PMC3960075

[ref38] Kalliokoski T.; Kramer C.; Vulpetti A.; Gedeck P. Comparability of Mixed IC50 Data – A Statistical Analysis. PLoS One 2013, 8, e61007 10.1371/journal.pone.0061007. 23613770 PMC3628986

[ref39] Todd B.; Tchesnokov E. P.; Götte M. The active form of the influenza cap-snatching endonuclease inhibitor baloxavir marboxil is a tight binding inhibitor. J. Biol. Chem. 2021, 296, 100486 10.1016/j.jbc.2021.100486. 33647314 PMC8065212

[ref40] Malik M. A.; Wani M. Y.; Al-Thabaiti S. A.; Shiekh R. A. Tetrazoles as carboxylic acid isosteres: chemistry and biology. J. Inclusion Phenom. Macrocyclic Chem. 2014, 78, 15–37. 10.1007/s10847-013-0334-x.

[ref41] Agrawal A.; DeSoto J.; Fullagar J. L.; Maddali K.; Rostami S.; Richman D. D.; Pommier Y.; Cohen S. M. Probing chelation motifs in HIV integrase inhibitors. Proc. Natl. Acad. Sci. U. S. A. 2012, 109, 2251–2256. 10.1073/pnas.1112389109. 22308350 PMC3289312

[ref42] Šebera J.; Dubankova A.; Sychrovský V.; Ruzek D.; Boura E.; Nencka R. The structural model of Zika virus RNA-dependent RNA polymerase in complex with RNA for rational design of novel nucleotide inhibitors. Sci. Rep. 2018, 8, 11132 10.1038/s41598-018-29459-7. 30042483 PMC6057956

