# Supplementary material for: Carboxylic Acid Isostere Derivatives of Hydroxypyridinones as Core Scaffolds for Influenza Endonuclease Inhibitors
Source: ACS Med Chem Lett. 2022 Dec 9;14(1):75–82. doi: 10.1021/acsmedchemlett.2c00434 (PMC9841593; doi:10.1021/acsmedchemlett.2c00434)
Supplement: Supplementary file 1 — ml2c00434_si_001.pdf [file ml2c00434_si_001.pdf]

Supporting Information

Carboxylic Acid Isostere Derivatives of Hydroxypyridinones as Core Scaffolds for Influenza Endonuclease Inhibitors

Ryjul W. Stokes,[‡a] *Alysia J. Kohlbrand,*[‡a] *Hyeonglim Seo,*[a] *Banumathi Sankaran,*[b] *Johannes Karges,*[a] *and Seth M. Cohen*[a*]

[a] Department of Chemistry and Biochemistry, University of California, San Diego, 9500 Gilman Drive, La Jolla, CA 92093, United States.

[b] The Berkeley Center for Structural Biology, Advanced Light Source, Lawrence Berkeley National Laboratory, Berkeley, California, 94720, United States

‡ Equal contribution

*scohen@ucsd.edu

Table of Contents

General Experimental Details	S3
Compound Synthesis	S4
PA_N Endonuclease Assay	S13
Computational Prediction of pK_a Values	S14
Comparative Predictions of cLogP	S15
Predicted Phenolic pK_a Values	S15
Protein Expression and Purification	S16
Protein Crystallography	S17
Protein Crystallography Tables	S19
Metal-binding Distances	S21
Octahedral Distortion Parameters	S21
Octahedral Distortion	S22
Representative HPLC Traces	S23
Lipophilic Ligand Efficiencies (LLE)	S23

General Experimental Details

All solvents and reagents, unless otherwise noted, were obtained from commercial sources and used without further purification. All reactions, unless otherwise stated, were performed under a nitrogen atmosphere and no unexpected or unusually high safety hazards were encountered. Silica chromatography was performed using a CombiFlash Rf Teledyne ISCO system using hexane (Hex), ethyl acetate (EtOAc), methylene chloride (DCM), or methanol (MeOH) as eluents. C_{18} reverse phase chromatography was performed using the same instrument using 0.1% formic acid in methanol, acetonitrile, or water as eluent. Several final compounds were further purified using a Gilson preparatory HPLC equipped with a UV detector, using Waters SunFire preparative C_{18} OBD columns (5 μM, 19×50 mm or 19×100 mm). A linear gradient from 10 to 90% MeCN in H_2O was used for 15 min with a flow rate of 20 mL/min. ^{1}H and ^{13}C NMR spectra were obtained on a Varian (400 MHz) spectrometer, Jeol (500 MHz) spectrometer or a VX (500 MHz) equipped with XSens cold probe (Varian) spectrometer in the Department of Chemistry and Biochemistry at U.C. San Diego. The purity of all compounds used in assays was determined to be ≥95% by HPLC analysis. Mass spectrometry was performed at the U.C. San Diego Molecular Mass Spectrometry Facility. HRMS analysis was performed using an Agilent 6230 Accurate-Mass LC-TOFMS located at the U.C. San Diego Molecular Mass Spectrometry Facility. pK_a values were determined using a Sirius T3 instrument. All titrations were performed in 0.15 M KCl with 0.5 M HCl and KOH. The pK_a values were determined by analyzing the MBI sample in triplicate using potentiometric titration. Experiments were performed over a pH range of 2.0–12.0.

Compound Synthesis

3-Hydroxy-4-oxo-1,4-dihydropyridine-2-carboxylic acid (**1**). 4-(Benzyloxy)-3-hydroxypicolinonitrile (200 mg, 0.88 mmol) was dissolved in 2.1 mL of a 5:5:1 mixture of concentrated hydrochloric acid, acetic acid, and trifluoro acetic acid. The reaction mixture was heated to 110 °C for 24 h. The solvent was removed in vacuo and the remaining residue was purified by C_{18} chromatography eluting in an H_2O/MeOH system to afford the product (12 mg, 9%). ^{1}H NMR (500 MHz, DMSO-d_6): δ 7.81 (d, J = 6.3 Hz, 1H), 7.02 (d, J = 6.4 Hz, 1H). ^{13}C NMR (126 MHz, DMSO-d_6): δ 164.6, 163.0, 152.0, 132.3, 126.5, 112.4. HR-ESI-MS experimental: 156.0292. Calculated for $[C_6H_6NO_4]^+$: 156.0291.

6-Bromo-3-hydroxy-4-oxo-1,4-dihydropyridine-2-carboxylic acid (**2**). 4-(Benzyloxy)-6-bromo-3-hydroxypicolinic acid (**2a**, 50 mg, 0.15 mmol) was dissolved in 5 mL of concentrated hydrochloric acid and stirred at room temperature for 30 h. The solvent was removed in vacuo and the remaining residue was taken up in water and extracted with ethyl acetate. The organic solvent was removed in vacuo and purified over C_{18} using H_2O/MeOH to afford the product (14 mg, 41 %). ^{1}H NMR (500 MHz, DMSO-d_6): δ 7.05 (s, 1H). ^{13}C NMR (126 MHz, DMSO-d_6): δ 170.7, 156.3, 149.5, 131.3, 129.9, 117.6. HR-ESI-MS experimental: 231.9252. Calculated for $[C_6H_3BrNO_4]^-$: 231.9251.

6-Bromo-3-hydroxy-4-oxo-1,4-dihydropyridine-2-carboxamide (**3**). KOH (184 mg, 3.28 mmol) and 4-(benzyloxy)-6-bromo-3-hydroxypicolinonitrile (**14**, 200 mg, 0.66 mmol) were combined in EtOH:H_2O (10 mL) and heated to 100 °C for 16 h. The reaction was allowed to cool to room temperature, and the solvent was removed in vacuo. The resulting residue was taken up in water and acidified with 4M HCl, resulting in the formation a white-yellow precipitate (**3a**). This solid was then dissolved in HCl and stirred for 48 h at room temperature. The reaction was dried in vacuo and purified over C_{18} using H_2O/MeOH to afford the product

(36 mg, 24 %). ^{1}H NMR (500 MHz, $(CD_3)_2CO$): δ 12.83 (s, 1H), 9.94 (s, 1H), 8.18 (s, 1H), 7.52 (s, 1H), 7.52, (s, 1H). ^{13}C (126 MHz, $(CD_3)_2CO$): δ 171.2, 155.5, 146.7, 130.6, 129.1, 117.4. HR-ESI-MS Experimental: 230.9411. Calculated for $[C_6H_4BrN_2O_3]^-$: 230.9411.

6-Bromo-3-hydroxy-2-(1H-tetrazol-5-yl)5yridine-4(1H)-one (**4**). 4-(Benzyloxy)-6-bromo-2-(1H-tetrazol-5-yl)pyridine-3-ol (**4a**) was taken directly from the previous step and dissolved in 5 mL of concentrated hydrochloric acid. The reaction mixture was heated to 100 °C for 8 h. Upon completion, the solvent was removed in vacuo and the resulting residue was purified over C_{18} using H_2O/MeOH to afford the product (33.4 mg, 40 %). ^{1}H NMR (500 MHz, CD_3OD): δ 7.07 (s, 1H). ^{13}C NMR (126 MHz, CD_3OD): δ 155.6, 149.9, 143.6, 131.5, 128.0, 115.8. HR-ESI-MS Experimental: 255.9475. Calculated for $[C_6H_3BrN_5O_2]^-$: 255.9476.

6-Bromo-3-hydroxy-N-methoxy-4-oxo-1,4-dihydropyridine-2-carboxamide (**5**). 6-Bromo-3-hydroxy-4-oxo-1,4-dihydropyridine-2-carboxylic acid (**2**, 42 mg, 0.18 mmol) was dissolved in 2 mL of thionyl chloride and heated to reflux for 12 h. The solvent was removed in vacuo and the resulting acid chloride was dissolved in 2 mL of DCM and cooled to -78 °C. Methoxyamine hydrochloride (29 mg, 0.35 mmol) and triethylamine (73 μL, 0.52 mmol) were added, and the reaction was warmed to r.t. and then heated to 40 °C for 48 h. The reaction mixture was filtered, and the filtrate was concentrated under reduced pressure. The remaining residue was purified over C_{18} using H_2O/MeOH to afford the product (15.7 mg, 34 %). ^{1}H NMR (500 MHz, CD_3OD): δ 7.04 (s, 1H), 3.82 (s, 3H). ^{13}C NMR (126 MHz, CD_3OD): δ 155.8, 149.9, 148.0, 129.9, 129.3, 117.1, 63.2. HR-ESI-MS Experimental: 260.9518. Calculated for $[C_7H_6BrN_2O_4]^-$: 260.9516.

6-Bromo-N',3-dihydroxy-4-oxo-1,4-dihydropyridine-2-carboximidamide (**6**). N'-acetoxy-3,4-bis(benzyloxy)-6-bromopicolinimidamide (**6a**, 100 mg, 0.21 mmol) was dissolved in 4 mL of concentrated hydrochloric acid. The solution was heated to 50 °C and stirred for 16 h. The

reaction was cooled to room temperature, and the solvent was removed in vacuo. The resulting residue was taken up in water and extracted with ethyl acetate, which was removed in vacuo to afford the product (41 mg, 78%). ^{1}H NMR (500 MHz, CD_3OD): δ 6.91 (s, 1H). ^{13}C NMR (126 MHz, CD_3OD): δ 154.9, 153.1, 143.9, 131.3, 129.1, 114.5. HR-ESI-MS Experimental: 247.9666. Calculated for $[C_6H_7BrN_3O_3]^+$: 247.9665.

3-(6-Bromo-3-hydroxy-4-oxo-1,4-dihydropyridin-2-yl)-1,2,4-oxadiazol-5(4H)-one (**7**). 3-(3,4-Bis(benzyloxy)-6-bromopyridin-2-yl)-1,2,4-oxadiazol-5(2H)-one (**7a**, 51 mg, 0.11 mmol) was dissolved in 4 mL of concentrated hydrochloric acid. The solution was heated to 50 °C and stirred for 16 h. The reaction was cooled to room temperature, and the solvent was removed in vacuo. The resulting residue was taken up in water and extracted with ethyl acetate. The organic layer was concentrated in vacuo and purified over C_{18}, using H_2O/MeOH as a gradient to afford the product (17 mg, 55%). ^{1}H NMR (500 MHz, CD_3OD): δ 7.03 (s, 1H). ^{13}C NMR (126 MHz, CD_3OD): δ 159.8, 157.0 155.5, 144.1, 131.0, 127.4, 115.8. HR-ESI-MS Experimental: 271.9312. Calculated for $[C_7H_3BrN_3O_4]^-$: 271.9312.

6-Bromo-3-hydroxy-2-(5-methyl-1,2,4-oxadiazol-3-yl)6yridine-4(1H)-one (**8**). N'-acetoxy-3,4-bis(benzyloxy)-6-bromopicolinimidamide (**8a**, 90 mg, 0.19 mmol) was dissolved in 11 mL of a 5:5:1 mixture of concentrated hydrochloric acid, acetic acid, and trifluoroacetic acid, and stirred for 48 h at 50 °C. The solvent was removed in vacuo and the product was purified over C_{18} using a H_2O/MeOH gradient (20 mg, 39%). ^{1}H NMR (500 MHz, CD_3OD): δ 7.06 (s, 1H), 2.74 (s, 3H). ^{13}C (126 MHz, CD_3OD): δ 177.5, 165.8, 155.9, 144.3, 131.4, 129.6, 115.9, 10.8). HR-ESI-MS Experimental: 271.9664. Calculated for $[C_8H_7BrN_3O_3]^+$: 271.9665

6-Bromo-3-hydroxy-4-oxo-1,4-dihydropyridine-2-carbonitrile (**9**). 4-(Benzyloxy)-6-bromo-3-hydroxypicolinonitrile (**14**, 100 mg, 0.33 mmol) was dissolved in 4 mL of concentrated hydrochloric acid and heated to 50 °C overnight. The solvent was removed in vacuo, and the

resulting solids were dissolved in water and extracted with DCM. The organic layer was dried in vacuo to afford the product (21 mg, 30 %). ^{1}H NMR (500 MHz, CD_3OD): δ 7.07 (s, 1H). ^{13}C NMR (126 MHz, CD_3OD): δ 155.5, 149.2, 130.9, 118.9, 116.5, 114.2. HR-ESI-MS Experimental: 212.9306. Calculated for $[C_6H_2BrN_2O_2]^-$: 212.9305.

6-Bromo-2-(4,5-dihydro-1H-imidazol-2-yl)-3-hydroxypyridin-4(1H)-one (**10**). 4-(Benzyloxy)-6-bromo-2-(4,5-dihydro-1H-imidazol-2-yl)7yridine-3-ol (**10a**, 77 mg, 0.22 mmol) was dissolved in 3 mL of concentrated hydrochloric acid and stirred overnight at room temperature. Upon completion, the solvent was removed, and the resulting residue was purified over C_{18} using H_2O/MeOH to afford the product (12 mg, 22 %). ^{1}H NMR (500 MHz, CD_3OD): δ 7.14 (s, 1H), 4.06 (s, 4H). ^{13}C NMR (126 MHz, CD_3OD): δ 162.0, 155.8, 147.3, 131.1, 125.4, 117.0, 44.2. HR-ESI-MS Experimental: 257.9871. Calculated for $[C_8H_9BrN_3O_2]^+$: 257.9873.

6-Bromo-2-(4,5-dihydrooxazol-2-yl)-3-hydroxypyridin-4(1H)-one (**11**). 4-(Benzyloxy)-6-bromo-2-(4,5-dihydrooxazol-2-yl)pyridin-3-ol (**11a**, 90 mg, 0.26 mmol) was dissolved in 4 mL of concentrated hydrochloric acid. The solution was heated to 50 °C and stirred for 16 h. The reaction was cooled to room temperature, and the solvent was removed in vacuo. The resulting residue was taken up in water and extracted with ethyl acetate. The organic layer was concentrated in vacuo and the resulting residue purified over C_{18}, using H_2O/MeOH as a gradient to afford the product (22 mg, 33 %). ^{1}H NMR (500 MHz, CD_3OD): δ 6.78 (s, 1H), 3.81 (t, 2H, J = 5.1 Hz), 3.52 (t, 2H, J = 5.1). ^{13}C NMR (126 MHz, CD_3OD): δ 161.2, 159.8, 159.7, 123.9, 122.9, 112.9, 59.4, 43.8. HR-ESI-MS experimental: 258.9718. Calculated for $[C_8H_8BrN_2O_3]^+$: 258.9713.

4-(Benzyloxy)-6-bromo-3-hydroxypicolinic acid (**2a**). 4-(Benzyloxy)-6-bromo-3-hydroxypicolinonitrile (**14**, 1.0 g, 3.3 mmol) was heated to 100 °C in Ethanol (20 mL) and 6.0 M NaOH (10 mL) for 20 h, during which, a white precipitate formed. The reaction was diluted

with 25 mL of water and 25 mL EtOAc were added. The solution was mixed and filtered. To the white precipitate was added 50 mL water, and the solution was acidified to pH ~2. The mixture was then filtered, affording 4-(benzyloxy)-6-bromo-3-hydroxypicolinic acid as a white solid in 83% yield. ^{1}H NMR (500 MHz, DMSO-d_6): δ 7.50 (s, 1H), 7.47 – 7.31 (m, 5H), 5.26 (s, 2H). ^{13}C NMR (126 MHz, DMSO-d_6): δ 170.7, 156.0, 149.8, 135.8, 130.7, 130.3, 129.0, 128.9, 128.7, 115.9, 71.2. HR-ESI-MS Experimental: 321.9717. Calculated for $[C_{13}H_9BrNO_4]^-$: 321.9720.

4-(Benzyloxy)-6-bromo-2-(1H-tetrazol-5-yl)pyridin-3-ol (**4a**). 4-(Benzyloxy)-6-bromo-3-hydroxypicolinonitrile (**14**, 100 mg, 328 μmol), sodium azide (32 mg, 492 μmol), and ammonium chloride (53 mg, 0.99 mmol) were combined in DMF (5 mL) and heated to 115 °C for 4 h. After the reaction mixture had cooled, it was poured into cool water (note: maintain the concentration of azide in solution <5%) and was acidified to pH 2 with 4M HCl. A white precipitate formed, and the solution was stirred in an ice bath for 30 min, after which time the solids were collected by filtration and dried to afford the product. The resulting solids were dissolved in EtOAc and washed several times with a 5% LiCl solution. Residual DMF was difficult to remove and did not affect the next step, which was undertaken immediately. ^{1}H NMR (500 MHz, DMSO-d_6): δ 7.54 – 7.50 (m, 3H), 7.45 – 7.41 (m, 2H), 7.39 – 7.35 (m, 2H), 5.35 (s, 2H). ^{13}C NMR (126 MHz, DMSO-d_6): δ 156.1, 144.2, 135.8, 131.4, 129.7, 129.0, 128.9, 128.7, 128.6, 114.4, 71.3. HR-ESI-MS Experimental: 345.9943. Calculated for $[C_{13}H_9BrN_5O_2]^-$: 345.9945.

4-(Benzyloxy)-6-bromo-N',3-dihydroxypicolinimidamide (**6a**). 4-(Benzyloxy)-6-bromo-3-hydroxypicolinonitrile (**14**, 1.0 g, 3.3 mmol), hydroxylamine hydrochloride (0.5 g, 6.6 mmol), and triethylamine (0.9 mL, 6.6 mmol) were combined in ethanol (10 mL) in a synth vial. The reaction mixture was heated to 50 °C for 1 h and cooled to room temperature, resulting in the formation of an off-white precipitate. The reaction mixture was diluted with water and

extracted with EtOAc two times. The addition of brine aided with emulsions. The organic layers were combined and concentrated under vacuum to afford the product (0.9 g, 90%). ^{1}H NMR (500 MHz, DMSO-d_6): δ 12.35 (s, 1H), 10.38 (s, 1H), 7.48 – 7.36 (m, 5H), 7.35 (s, 1H), 6.30 (s, 2H), 5.22 (s, 2H). ^{13}C NMR (126 MHz, DMSO-d_6): δ 155.5, 153.1, 145.1, 136.1, 131.7, 129.3, 129.0, 128.8, 128.7, 113.1, 70.9. HR-ESI-MS Experimental: 335.9989. Calculated for $[C_{13}H_{11}BrN_3O_3]^-$: 335.9989.

3-(3,4-Bis(benzyloxy)-6-bromopyridin-2-yl)-1,2,4-oxadiazol-5(4H)-one (**7a**). 3,4-Bis(benzyloxy)-6-bromo-N'-hydroxypicolinimidamide (**16**, 200 mg, 0.47 mmol), CDI (151 mg, 0.93 mmol), and triethylamine (78.1 μL, 0.56 mmol) were dissolved in 5 mL of DMF. The reaction mixture was heated to 80 °C for 8 h. The solvent was removed in vacuo and ethyl acetate was added. The solution was rinsed with water and the organic solvent removed in vacuo to afford the product (142 mg, 67 %). ^{1}H NMR (500 MHz, $CDCl_3$): δ 7.45 (s, 5H), 7.38 – 7.36 (m, 2H), 7.33 – 7.30 (m, 3H), 7.21 (s, 1H), 5.21 (s, 2H), 5.16 (s, 2H). HR-ESI-MS Experimental: 452.0258. Calculated for $[C_{21}H_{15}BrN_3O_4]^-$: 452.0251

4-(Benzyloxy)-6-bromo-2-(4,5-dihydro-1H-imidazol-2-yl)pyridin-3-ol (**10a**). 4-(Benzyloxy)-6-bromo-3-hydroxypicolinonitrile (**14**, 100 mg, 0.33 mmol) was dissolved in toluene (3 mL), followed by the addition of ethane-1,2-diamine (29.5 mg, 0.30 μL, 0.49 mmol) and zinc chloride (0.6 mg). The reaction was heated to 130 °C for 20 h. Upon completion, the reaction was allowed to cool to room temperature and the solvent was removed in vacuo. Ethyl acetate was added, and solution was washed with water. The organic solvent was removed in vacuo and the resulting residue purified over silica using a 0-10% (DCM/MeOH) gradient to afford the product (77 mg, 68%). ^{1}H NMR (500 MHz, $CDCl_3$): δ 7.43 (d, J = 7.3 Hz, 2H), 7.36 (t, J = 7.3 Hz, 2H), 7.34 – 7.31 (m, 1H), 7.27 (s, 1H), 5.09 (s, 2H), 3.92 – 3.82 (m, 4H). HR-ESI-MS experimental: 348.0342. Calculated for $[C_{15}H_{15}BrN_3O_2]^+$: 348.0342.

4-(Benzyloxy)-6-bromo-2-(4,5-dihydrooxazol-2-yl)pyridin-3-ol (**11a**). 4-(Benzyloxy)-6-bromo-3-hydroxypicolinonitrile (**14**, 100 mg, 0.33 mmol) was dissolved in toluene (2 mL), followed by the addition of 2-aminoethan-1-ol (29.7 μL, 0.49 mmol) and zinc chloride (0.6 mg). The reaction mixture was heated to 90 °C for 16 h. Upon completion, the reaction was allowed to return to room temperature and the solvent was removed in vacuo. The remaining residue was taken up in ethyl acetate and washed with water. The organic layer was concentrated to afford the product (96 mg, 84%). ^{1}H NMR (500 MHz, CD_3OD): δ 7.51 – 7.47 (m, 2H), 7.40 – 7.34 (m, 2H), 7.34 – 7.29 (m, 1H), 6.92 (s, 1H), 5.13 (s, 2H), 3.79 (t, J = 5.1 Hz, 2H), 3.50 (t, J = 5.1 Hz, 2H). HR-ESI-MS experimental: 349.0183. Calculated for $[C_{15}H_{14}BrN_2O_3]^+$: 349.0182.

4,6-Dibromo-3-hydroxypicolinonitrile (**13**). Compound **13** was prepared as previously described (*Patent WO2016007634,* **2016).** To a mechanically stirred solution of 3-hydroxypicolinoitrile (**12**, 10.0 g, 83.3 mmol) and sodium acetate (13.7 g, 167 mmol) in water (150 mL) and methanol (50 mL) at 0 °C was slowly added Br_2 (13 mL, 250 mmol) from a pressure-equalizing dropping funnel while maintaining the temperature at <20 °C. The reaction mixture was then stirred overnight at room temperature. After the reaction was complete, as indicated by TLC analysis, the reaction mixture was cooled to 5-10 °C, and slowly charged with an aqueous solution of 25% $NaHSO_3$ (100 mL) while keeping the temperature at <20 °C. The resulting suspension was stirred for 30 min and filtered. The filter cake was washed with water, dried in air for several hours and then under vacuum to afford the product as a light-yellow solid (21.5 g, 93 %). ^{1}H NMR (500 MHz, DMSO-d_6): δ 8.27 (s, 1H). ^{13}C NMR (126 MHz, DMSO-d_6): δ 156.3, 136.2, 130.0, 126.6, 122.0, 115.2. HR-ESI-MS experimental: 274.8462. Calculated for $[C_6HBr_2N_2O]^-$: 274.8461.

4-(Benzyloxy)-6-bromo-3-hydroxypicolinonitrile (**14**). Compound **14** was prepared as previously described (*J. Med. Chem.* **2019**, *62,* 9438-9449). In a flame-dried flask, NaH (3.6 g, 90 mmol) was stirred in dry DMSO (20 mL, dried on molecular sieves). Benzyl alcohol (9.4 mL, 90 mmol) was added dropwise to the mixture, pausing when excessive foam formed. When the reaction was complete, as evidenced by the cessation of hydrogen evolution, 4,6-dibromo-3-hydroxypicolinonitrile (**13**, 5.0 g, 18 mmol) in dry DMSO (10 mL) was added dropwise to the stirring solution, and the mixture was heated to 60 °C for 18 h, monitoring conversion by thin-layer chromatography. When the reaction was complete, the mixture was placed in an ice bath, and 150 mL of water were added. The mixture was stirred for 10 min, during which time the solution clouded and then clarified. To remove residual benzyl alcohol, the aqueous solution was extracted twice with 100 mL portions of diethyl ether. Residual ether was then removed from the aqueous portion under vacuum, and the solution was cooled to 5 °C on ice. The addition of 4M HCl (until pH ~2) resulted in a solid precipitate. It is imperative to maintain an ice bath temperature during acidification; otherwise, excess heat or residual ether present in the solution will result in the formation of a gummy, orange oil in lieu of off-white crystallites. If an oil forms, it can be slowly dissolved in basic (pH > 10) water with sonication to repeat the precipitation. The acidified mixture was stirred for 30 min on ice, and the resultant solids were filtered, rinsed with H_2O, and dried. Purification via silica gel chromatography in an EtOAc/Hex system afforded 4-(benzyloxy)-6-bromo-3-hydroxypicolinonitrile (4.3 g, 78%). ^{1}H NMR (500 MHz, DMSO-d_6): δ 11.62 (s, 1H), 7.57 (s, 1H), 7.53 – 7.48 (m, 2H), 7.44 – 7.34 (m, 3H), 5.33 (m, 2H). HR-ESI-MS experimental: 302.9777. Calculated for $[C_{13}H_8BrN_2O_2]^-$: 302.9775.

3,4-Bis(benzyloxy)-6-bromopicolinonitrile (**15**). 4-(Benzyloxy)-6-bromo-3-hydroxypicolinonitrile (**14**, 1.0 g, 3.3 mmol) and potassium carbonate (0.9 g, 6.6 mmol) were taken up in DMF (30 mL), and (bromomethyl)benzene (0.8 mL, 6.6 mmol) was added slowly.

The reaction was stirred for 19 h at 40 °C. After cooling, 30 mL of ethyl acetate was added to the reaction mixture, which was then washed with 30 mL of water. The organic layer was collected, and the aqueous layer was extracted once more with 30 mL of ethyl acetate. The organic layers were combined and concentrated in vacuo. The product was separated over silica using a 0-50% Hex/EtOAc gradient. (1.2 g, 91%). ^{1}H NMR (500 MHz, DMSO-d_6): δ 7.83 (s, 1H), 7.55 – 7.50 (m, 2H), 7.48 – 7.41 (m, 3H), 7.35 – 7.32 (m, 5H), 5.38 (s, 2H), 5.22 (s, 2H). ^{13}C NMR (126 MHz, DMSO-d_6): δ 159.8, 148.8, 136.9, 135.8, 135.3, 129.3, 129.2, 128.9, 128.8, 126.8, 117.6, 114.8, 75.9, 72.1. HR-ESI-MS experimental: 395.0387. Calculated for $[C_{20}H_{16}BrN_2O_2]^+$: 395.0390.

3,4-Bis(benzyloxy)-6-bromo-N'-hydroxypicolinimidamide (**16**). 3,4-Bis(benzyloxy)-6-bromopicolinonitrile (**15**, 2.1 g, 5.0 mmol), hydroxylamine hydrochloride (0.9 g, 13.2 mmol), and triethylamine (1.8 mL, 18.8 mmol) were combined in Ethanol (200 mL) in a round-bottom flask. The reaction mixture was heated to 50 °C for 15 h. The solvent was removed in vacuo and the residue was taken up in ethyl acetate and rinsed with water. The organic layer was collected and removed in vacuo to afford the product (2.1 g, 97 %). ^{1}H NMR (500 MHz, $(CD_3)_2CO$: δ 9.34 (s, 1H), 7.08 – 7.01 (m, 3H), 7.01 – 6.89 (m, 5H), 6.87 – 6.81 (m, 3H), 5.31 (s, 2H), 4.85 (s, 2H), 4.51 (s, 2H). HR-ESI-MS experimental: 428.0599. Calculated for $[C_{20}H_{19}BrN_3O_3]^+$: 428.0604.

N'-acetoxy-3,4-bis(benzyloxy)-6-bromopicolinimidamide (**17**). To a 5 ºC solution of 4-(benzyloxy)-N',3-dihydroxypicolinimidamide (**16**, 100 mg, 0.23 mmol) and DBU (77.4 μL, 0.51 mmol) in dry THF stirring on ice was added acetic anhydride (44.1 μL, 0.47 mmol) dropwise. The solution was stirred 30 min on ice and was then heated to 80 ºC for 8 h. The solvent was then evaporated in vacuo. The resulting mixture was dissolved in ethyl acetate and rinsed with water. The organic layer was concentrated in vacuo and the resulting residue was separated over silica using Hex/EtOAc as a gradient to afford the product (110 mg, 80%). ^{1}H

NMR (500 MHz, $CDCl_3$): δ 7.46 – 7.43 (m, 2H), 7.41 (s, 5H), 7.29 – 7.25 (m, 3H), 7.13 (s, 1H), 5.64 – 5.54 (bs, 2H), 5.15 (s, 2H), 5.14 (s, 2H), 2.22 (s, 3H). HR-ESI-MS experimental: 470.0707. Calculated for $[C_{22}H_{21}BrN_3O_4]^-$: 470.0710.

PA_N Endonuclease Assay.

PA_N endonuclease activity assays were carried out as previously reported (*J. Med. Chem.* **2016**, *59*, 6444-6454). Assays were performed using Black Costar 96-well plates. Each well contained a total volume of 100 μL comprised of buffer (20 mM Tris, 150 mM NaCl, 2 mM $MnCl_2$, 0.2% TritonX100, pH 8.0, 0.2% 2-mercaptoethanol), influenza PA_N endonuclease (25 nM), inhibitor (various concentrations), and fluorescent ssDNA-oligo substrate (250 nM). A single-stranded, 17-mer DNA substrate labeled with a 5′-FAM fluorophore and a 3′-TAMRA quencher ([6-FAM]AATCGCAGGCAGCACTC[TAM], Sigma-Aldrich) was employed as the substrate. All assay components were pipetted into the plate, and ultimately, the substrate was added using a multi-channel pipette, and the assay was immediately started. Samples were prepared in triplicates. Background wells consisting of all assay components except enzyme were prepared for each sample. Positive and negative controls were prepared on each plate to gauge the fluorescence signal of fully active protein and the absence of protein. Change in fluorescence of each well was measured by a Synergy H4 Hybrid Multi-Mode Microplate Reader (BioTek) at 39 second intervals over 45 min at 37 °C (λ_{ex} = 485 nm; λ_{em} = 528 nm). The gain was set to 100. Typically, data collected between 20 and 35 min was used in the activity calculations, as this data range had a linear slope. The slope of the fluorescence signal for each sample was background corrected and percent inhibition was determined by normalizing the slope of the sample to that of the positive and negative controls. Dose response curves were generated for inhibitors by plotting percent inhibition versus log of the concentration for each inhibitor. The data were fit with a sigmoidal curve to determine the IC_{50} value using a four parameter MATLAB script. Ligand efficiency (LE) values were calculated using the following

equation: LE = ΔG/heavy atom count (HAC). This equation can be calculated for IC_{50} values using the equation: -RTln(IC_{50})/HAC, which can be approximated as 1.4(pIC_{50})/HAC (*Drug Discovery Today* **2005**, *10*, 987-992).

Computational Prediction of pK_a Values

The predictions were performed slightly modified from a published procedure (*Chem. Phys. Lett.* **2003,** *367*, 145-149). Density functional theory (DFT) calculations were performed with the Gaussian software package (Gaussian, Inc., Wallingford CT, 2016). The calculations were done with the B3LYP hybrid functional and the 6-31++ g(d,p) basis set. Solvent effects were included using CPCM model. The concentration of bulk water was considered to be 55.49 M. The structures of all calculated molecules correspond to ground state minima on the ground state potential energy surfaces with no imaginary frequencies present. The following thermodynamic cycle and equations were used to determine the pK_a value:

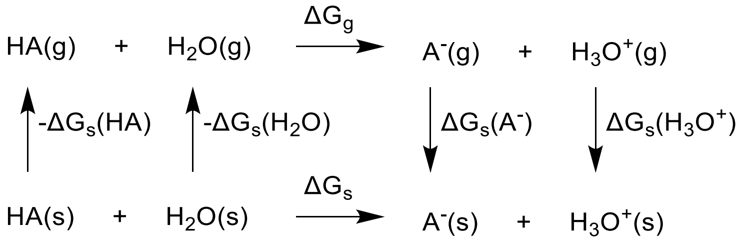

s=aqueous phase, g=gas phase

$$\Delta G_s = \Delta G_g + \Delta G_s(A^-) + \Delta G_s(H_3O^+) - \Delta G_s(HA) - \Delta G_s(H_2O)$$

$$pK_a = (\Delta G_s / 1.364) - \log(H_2O)$$

Table S1. Comparative predictions of cLogP calculated using MarvinSketch 19.6.

**Compound**	R = H	R = Br	R = Me	R = Ph
**2-R**	-0.35	0.47	-0.14	0.95
**3-R**	-1.15	-0.33	-0.95	0.15
**4-R**	-1.04	-0.22	-0.84	0.26
**5-R**	-0.78	0.04	-0.58	0.52
**6-R**	-1.09	-0.27	-0.88	0.21
**7-R**	-0.31	0.5	-0.11	0.98
**8-R**	-0.10	0.72	0.05	0.88
**9-R**	-0.12	0.70	0.08	1.18
**10-R**	-0.75	0.70	-0.55	0.55
**11-R**	0.07	0.88	0.27	1.36

Table S2. Predicted and measured phenolic pK_a values.

**Compound**	**Calculated pK_a**	**Measured pK_a**
**1**	8.5	-
**2**	9.4	9.79 ± 0.01
**3**	9.2	9.64 ± 0.02
**4**	12.6	>12
**5**	8.4	-
**6**	8.9	9.06 ± 0.02
**7**	9.8	-
**8**	10.7	-
**9**	8.1	-
**10**	9.7	-
**11**	9.5	-

Protein Expression and Purification

Expression and purification of PA_N endonuclease was performed as reported previously (*J. Med. Chem.* **2019,** *62*, 9438-9449). The pandemic isolate A/California/04/2009(H1N1) N-terminal PA (PA_N) endonuclease Δ52-64:Gly truncated construct was expressed from a pET-28a parent vector containing a kanamycin-resistance reporter gene with expression inducible by the Lac1 operon. PA_N endonuclease was expressed as an 8-histidine tagged fusion protein cleavable by TEV protease. The transformation protocol was adapted from pET system manual (Novagen) using single competent BL21 cells. Briefly, 1 µL of 25 ng/µL recombinant plasmid was used for transformation. Cells were mixed with the plasmid and were heat shocked at 42 °C for 30 sec followed by incubation on ice for 3 min. Outgrowth was plated on LB agarose plates containing 50 µg/mL kanamycin and was incubated overnight at 37 °C. One colony was scraped from the LB plate and added to 5 mL of SOC broth containing 50 µg/mL kanamycin and was incubated overnight at 37 °C with shaking at 125 rpm. SOC media (100 mL) containing 50 µg/mL kanamycin was combined with the 5 mL overnight growth and was incubated with shaking at 200 rpm at 37 °C until the OD_{600} of this starter culture reached >2 (3-4 hours). The culture was equally divided into 6, 2L flasks containing 1L of expression media (TB media with added 0.2% dextrose, 0.1 mM $MnCl_2$, and 0.1 mM $MgSO_4$, 50 µg/mL kanamycin). Cells were grown to and OD_{600} between 0.4-0.6 at room temperature with shaking at 200 rpm (3-4 hours). Expression was then induced by addition of IPTG to a final concentration of 0.1 mM. The cultures were grown with vigorous shaking (250 rpm) overnight at room temperature. After ~18 h the cells were harvested by centrifuging at 2000g for 30 min at 4 °C. The resulting paste was stored at -80 °C prior to lysis.

Cell paste was thawed in batches on ice for 2 h and resuspended in 25-35 mL of lysis buffer (1% Triton-X, 1mM $MgCl_2{\cdot}6H_2O$, 2mM DTT, 10-100 µg/mL DNAse-1, 1 mg/ml lysozyme, and 1% glycerol) and EDTA free protease inhibitor (Roche). Cells were lysed using a probe sonicator (Fisherbrand model 120) with cycles of 25 second pulses and 59 second rest at 60% amplitude. Cell debris was then pelleted by centrifugation at 10000 rpm for 45 min at 4 °C. The supernatant was decanted from the pellet, and a HisTrap FF (Cytiva) column was utilized to isolate His-tagged fusion protein from the cell lysates according to the manufacturer's recommendations at 4 °C. Briefly, cell-free lysates from 6 L growth were loaded on 5 mL column that had previously been charged with Ni ions. The column was then washed with binding buffer (20 mM Na_2PO_4, 500 mM NaCl, 25 mM imidazole, pH 7.4) until fraction absorbance reached a steady baseline. The protein was then eluted over a gradient from 0-100%

elution buffer (20 mM Na_2PO_4, 500 mM NaCl, 500 mM imidazole, pH 7.4) at a flow rate of 4 mL/min. PA_N endonuclease eluted between 40-60% elution buffer. SDS-PAGE analysis showed a band corresponding to PA_N endonuclease running at ~23 kDa with several small impurities.

Fractions containing PA_N endonuclease were combined in a 10K MWCO dialysis bag with 1000 units of TEV protease and were dialyzed against dialysis buffer (100 mM NaCl, 1 mM dithiothreitol, 1 mM $MnCl_2$, 20 mM Tris, 5% glycerol, pH 8.0) overnight with three buffer exchanges. The proteolytic cleavage of the fusion protein is slow and greatly benefits from the addition of excess TEV protease. A white precipitate forms over time. After buffer exchange, the solution was filtered through a 0.45 μm filter. The solution was run through the Histrap FF column equilibrated with the same binding buffer as before. The resulting flow through contained His-cleaved PA_N endonuclease, which was then concentrated to 5-10 mg/mL using a pressurized Amicon and/or spin Amicon concentrator. The concentrated protein was then purified on a gel-permeation size exclusion column (GE Superdex 75, 10/300 GL) according to manufacturer recommendations in buffer (150 mM NaCl, 2 mM $MgCl_2$, 2 mM $MnCl_2$, 20 mM HEPES, pH 7.5). A large peak corresponding to the cleaved PA_N endonuclease eluted at ~12 mL eluent. A small shoulder before the main peak was occasionally observed, which contained primarily uncleaved and/or unfolded PA_N endonuclease construct. Fractions containing pure cleaved PA_N endonuclease were combined and concentrated to 2-5 mg/mL. Stored protein was flash-frozen in liquid nitrogen and was kept at -80 °C. This protein was suitable for use in enzyme or thermal shift assays or for protein crystallography.

Protein Crystallography

Purified protein for crystallization was stored at 2.2-4.3 mg/mL at -80 °C after flash freezing in buffer consisting of 150 mM sodium chloride, 20 mM HEPES (pH 7.5), 2 mM $MgCl_2$, and 2 mM $MnCl_2$. Co-crystallization and crystal soaking methods were used to obtain co-crystal structures of inhibitors bound to PA_N endonuclease. For co-crystallization, protein was incubated with 0.5 mM inhibitor for 1 hour on ice prior to setting the crystallization drops. For crystal soaking, fully formed holo crystals were transferred to a new drop containing 5 μL of reservoir solution and 1 μL of 50 mM DMSO inhibitor stock solution (final concentration 8.3 mM). Crystals were left undisturbed overnight and either stored in liquid nitrogen, or collected on an in-house X-ray diffractometer the following day. In both crystallization methods, crystals were grown using hanging drop and set in 24-well pre-greased plates (Hampton HR3-171) with

siliconized glass slides (Hampton HR3-231). A 5:1 ratio of purified protein to reservoir solution at room temperature was found to be the optimal ratio for the largest crystal formation. Reservoir solution consisted of 22-34% PEG (MW 4000 g/mol), 100 mM Tris (pH 8.35), and 220 mM sodium acetate. Colorless crystals with hexagonal bipyramidal morphology appeared within 2 days and reached full size after 1-2 weeks. Crystals were typically 50 to 200 microns in diameter. Crystals were cryoprotected with perfluoroether (Hampton HR2-814) prior to flash freezing in liquid nitrogen. Crystals were stored in liquid nitrogen until data collection.

Datasets for protein co-crystallized with compounds **5, 9,** and **10** were collected on an in- house X-ray diffractometer. For these experiments, diffraction data was collected at 100 K on a Bruker X8 Proteum diffractometer using a Bruker Microfocus Rotating Anode (MicroStar FR-592) X-ray generator with a Bruker APEX II CCD detector at wavelength 1.54178 Å. Data was integrated, scaled, and merged using the Bruker APEX3 software package (Bruker, 2017). Datasets for protein co-crystallized with compounds **1, 2, 3, 4,** and **8** were collected at the Advanced Light Source (ALS), Lawrence Berkeley National Laboratory, in collaboration with Dr. Banu Sankaran through Collaborative Crystallography program on beamline 8.2.1. For all datasets (including datasets collected on the in-house X-ray generator), phasing was determined by molecular replacement against a previously published PA_N endonuclease structure (PDB 4AWM) using PHASER. All structures were refined with Phenix version 1.19.2.

Table S3. X-ray crystallographic data collection and refinement statistics

Compound	**1**	**2**	**3**	**4**
**PDB**	8CTF	8DDB	7V04	8DDE
**Data collection statistics**				
Resolution range	57.45 - 2.14 (2.217 - 2.14)	58.02 - 2.15 (2.227 - 2.15)	65.32 - 1.91 (1.978 - 1.91)	57.81 - 2.22 (2.299 - 2.22)
Space group	$P6_222$	$P6_222$	$P6_222$	$P6_222$
Cell dimensions a, b, c, (Å) α, β, γ (°)	75.66 75.66 119.451 90 90 120	76.228 76.228 121.608 90 90 120	75.421 75.421 119.317 90 90 120	75.979 75.979 121.005 90 90 120
Unique reflections	11738 (1134)	11965 (1158)	16216 (1580)	10783 (1053)
Completeness (%)	99.65 (99.74)	99.91 (99.83)	99.86 (100.00)	99.81 (99.15)
Mean I/sigma(I)	23.37 (2.50)	25.56 (2.58)	29.60 (2.59)	24.87 (2.54)
R-merge	0.07424 (1.636)	0.0699 (2.003)	0.05714 (1.42)	0.08323 (2.05)
R-measured	0.07587 (1.669)	0.07145 (2.043)	0.05841 (1.455)	0.08492 (2.092)
R-work	0.2030 (0.3203)	0.1824 (0.2868)	0.1967 (0.3112)	0.1876 (0.3225)
R-free	0.2419 (0.3374)	0.2385 (0.3637)	0.2347 (0.3702)	0.2442 (0.4187)
RMS(bonds)	0.003	0.011	0.008	0.01
RMS(angles)	0.44	1.1	0.98	1.02
Ramachandran favored (%)	98.29	94.86	98.86	94.89
Ramachandran outliers (%)	0	0.57	0	0.57
Average B-factor	68.07	68.88	51.24	64.51
Redundancy	25	25	24.4	26.2
CC1/2	1.000	0.999	0.999	1.000

* Metrics for highest resolution shell given in parentheses

Table S4. X-ray crystallographic data collection and refinement statistics.

Compound	**5**	**8**	**9**	**11**
**PDB**	8DJV	8DHN	8DAL	8DJY
**Data collection statistics**				
Resolution range	37.24-2.08 (2.15-2.08)	64.98-2.40 (2.48-2.40)	40.15-2.2 (2.28-2.2)	44.29-2.5 (2.5-2.59)
Space group	$P6_222$	$P6_222$	$P6_222$	$P6_222$
Cell dimensions a, b, c, (Å) α, β, γ (°)	74.465 74.465 120.259 90 90 120	75.034 75.034 121.24 90 90 120	75.403 75.403 120.436 90 90 120	75.807 75.807 119.978 90 90 120
Unique reflections	12322 (1077)	15004 (1221)	11981 (1049)	13372 (732)
Completeness (%)	99.1 (90.8)	99.74 (100.0)	100.0 (100.0)	100.0 (100.0)
Mean I/sigma(I)	13 (2.3)	20 (2.0)	17.2 (1.5)	20 (2.0)
R-merge	0.1 (0.775)	0.1 (1.439)	0.09 (1.368)	0.091 (1.243)
R-measured	0.103 (0.815)	0.102 (1.478)	0.092 (1.432)	0.093 (1.292)
R-work	0.2626 (0.3450)	0.2006(0.2792)	0.1998 (0.3086)	0.2116 (0.3358)
R-free	0.291 (0.354)	0.2563 (0.3205)	0.2576 (0.3866)	0.2860 (0.3964)
RMS(bonds)	0.01	0.004	0.004	0.008
RMS(angles)	1.21	0.62	0.57	0.86
Ramachandran favored (%)	94.32	96.57	98.86	96.02
Ramachandran outliers (%)	0	0	0	0
Average B-factor	48.6	54.1	54.5	57.6
Redundancy	17.7	29	19.6	19.1
CC1/2	0.999	1.000	1.000	0.999

* Metrics for highest resolution shell given in parentheses

Table S5. Metal-binding distances between coordinating atoms and metals. IC_{50} values are shown for context.

**Compound**	**Isostere-Mn_2**	**OH-Mn_2**	**OH-Mn_1**	**Carbonyl-Mn_1**	**Mn-Mn**	**IC_{50}**
**1**	2.2 Å	1.9 Å	2.3 Å	1.7 Å	3.9 Å	0.05 µM
**2**	2.1 Å	2.1 Å	2.2 Å	2.2 Å	3.7 Å	0.07 µM
**3**	2.1 Å	2.0 Å	2.2 Å	2.2 Å	3.8 Å	0.09 µM
**4**	1.8 Å	2.3 Å	1.9 Å	2.3 Å	3.7 Å	0.09 µM
**5**	2.0 Å	2.2 Å	2.1 Å	2.0 Å	3.8 Å	0.32 µM
**8**	2.1 Å	2.3 Å	2.1 Å	2.1 Å	3.9 Å	0.12 µM
**9**	2.8 Å	2.2 Å	2.2 Å	2.3 Å	3.9 Å	0.40 µM
**10**	2.0 Å	2.3 Å	2 Å	2.3 Å	3.8 Å	>0.5 µM

Table S6. Octahedral Distortion Parameters.

**Compound**	**D_{mean}**	**ζ**	**Σ**
Ligand free (water)	2.1914	0.685765	44.5718
**3**	2.1662	0.357853	46.6692
**4**	2.1378	1.151002	64.0144
**8**	2.2385	0.574277	90.8137
**8** (artificially forced planar)	2.2330	0.606847	98.3470
**9**	2.3006	1.042725	92.6387

Table S7. Lipophilic Ligand Efficiencies (LLE) calculated according to the method described in *Nat. Rev. Drug Discovery* **2014**, *13*, 105-121.

**Compound**	**1**	**2**	**3**	**4**	**5**	**6**	**7**	**8**	**9**	**10**	**11**
**LLE**	6.37	6.32	6.23	6.35	5.84	5.62	6.71	6.26	5.59	-	-

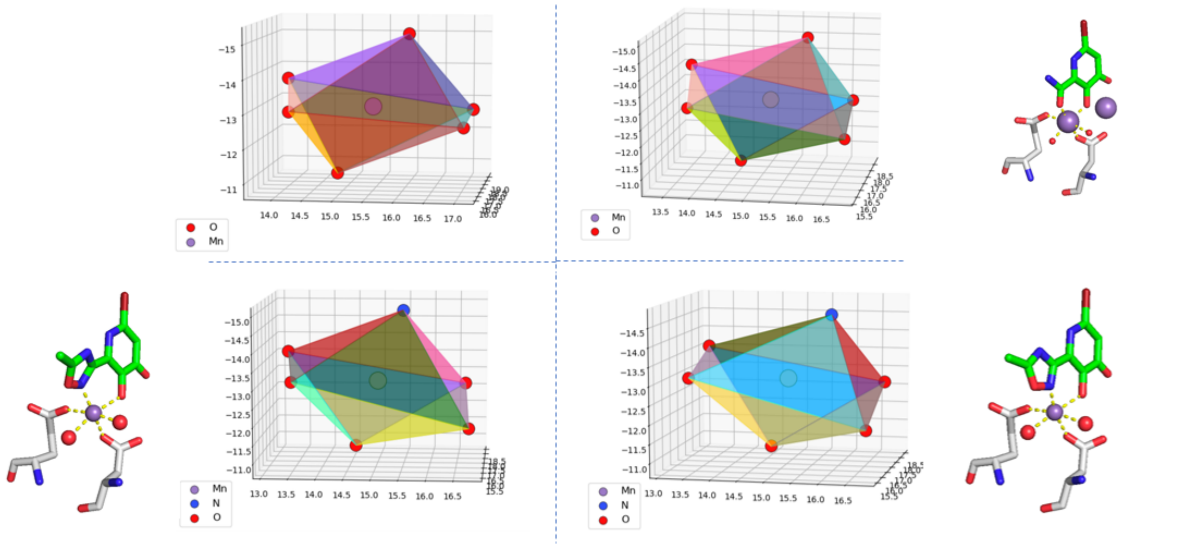

Figure S1. Visualization of octahedral distortion around Mn2 in PA_N endonuclease. The ligand coordination sphere is shown with the central purple sphere representing Mn2, red spheres representing oxygen donor atoms, and blue spheres representing nitrogen donor atoms. *Top left*: Octahedron of Mn2 with no inhibitor (bound water molecules), *Top right*: Octahedron of experimentally measured structure of compound **3**. *Bottom left*: Octahedron of experimentally measured structure of compound **8**. *Bottom right*: An 'artificial structure' of compound **8** forced into a planar conformation. Octahedral distortion parameters are shown in Table S6.

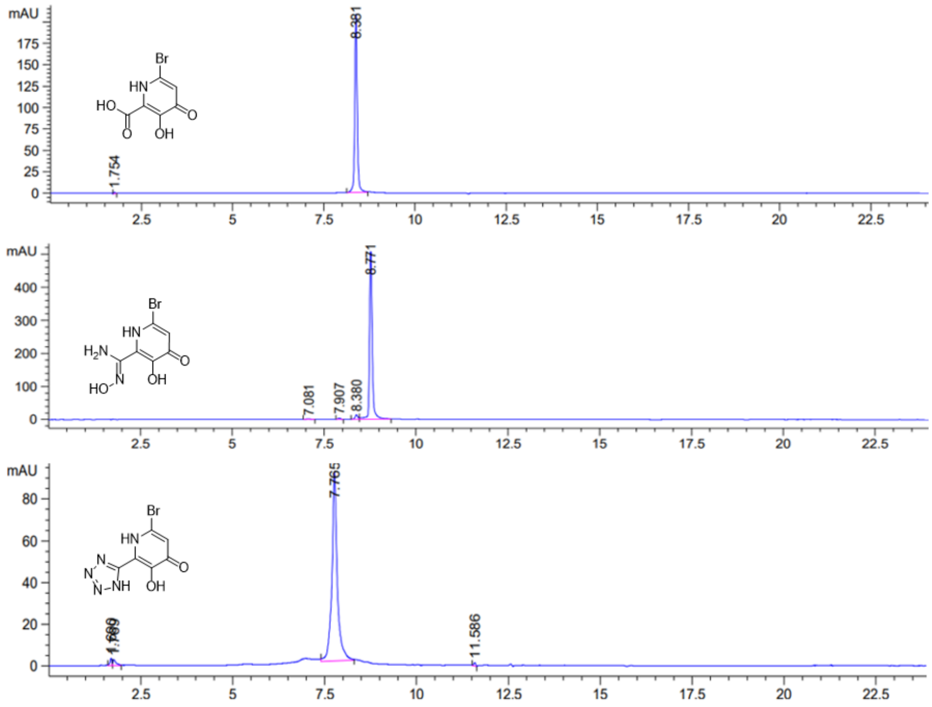

Figure S2. Representative HPLC traces for compounds **2**, **4**, and **6**.
